# Artificial intelligence models for pre-travel consultation and advice: yea or nay?

**DOI:** 10.1093/jtm/taad124

**Published:** 2023-10-03

**Authors:** Jinghao Nicholas Ngiam, Matthew Chung Yi Koh, Priscillia Lye, Tze Sian Liong, Brenda Mae Alferez Salada, Paul Anantharajah Tambyah, Jolene Ee Ling Oon

**Affiliations:** Division of Infectious Diseases, Department of Medicine, National University Health System, Singapore, 1E Kent Ridge Rd, Singapore 119228, Singapore; Division of Infectious Diseases, Department of Medicine, National University Health System, Singapore, 1E Kent Ridge Rd, Singapore 119228, Singapore; Division of Infectious Diseases, Department of Medicine, National University Health System, Singapore, 1E Kent Ridge Rd, Singapore 119228, Singapore; Department of Medicine, National University Health System, Singapore, 1E Kent Ridge Rd, Singapore 119228, Singapore; Division of Infectious Diseases, Department of Medicine, National University Health System, Singapore, 1E Kent Ridge Rd, Singapore 119228, Singapore; Yong Loo Lin School of Medicine, National University of Singapore, Singapore, 10 Medical Drive, Singapore 117597, Singapore; Division of Infectious Diseases, Department of Medicine, National University Health System, Singapore, 1E Kent Ridge Rd, Singapore 119228, Singapore; Yong Loo Lin School of Medicine, National University of Singapore, Singapore, 10 Medical Drive, Singapore 117597, Singapore; Infectious Diseases Translational Research Programme, Department of Medicine, Yong Loo Lin School of Medicine, National University of Singapore, Singapore, 10 Medical Drive, Singapore 117597, Singapore; Division of Infectious Diseases, Department of Medicine, National University Health System, Singapore, 1E Kent Ridge Rd, Singapore 119228, Singapore; Yong Loo Lin School of Medicine, National University of Singapore, Singapore, 10 Medical Drive, Singapore 117597, Singapore

**Keywords:** Artificial intelligence, ChatGPT, pre-travel consultation, vaccination

ChatGPT is an artificial intelligence (AI) chatbot that creates conversational dialogue with the user.^1^ It has been used in clinical care and medical education.^2^ Its potential role in diabetes education for patients has been explored, but safety concerns have been raised.^3^^,^^4^ There has been much interest in how AI can have a role in Travel Medicine in teaching, providing more personalized advice, and aid in the pre-travel consultation.^5^^,^^6^

Pre-travel consultation and advice is often provided by primary care providers and travel medicine specialists.^7^ It is an effective tool that can reduce the morbidity of traveller’s diarrhoea and improve health-seeking behaviour, although its uptake has been dismal.^8^^,^^9^ With increasing accessibility and widespread use of ChatGPT, we anticipate that travellers may turn to chatbots as a convenient option to seek pre-travel advice. As such, we aimed to evaluate pre-travel advice provided by ChatGPT.

We instructed ChatGPT to give pre-travel advice and subsequently provided the application with a series of commonly-asked questions, pertaining to general travel queries (such as food and water safety, sexual health, traveller’s diarrhoea), vaccinations and malaria prophylaxis. The accuracy and appropriateness of the responses were then compared against recommendations from the Centers for Disease Control Yellow Book 2024 and Shoreland Travax.^10^

ChatGPT could answer all the questions provided. The answers given were concise and often in point form, making them easy to read and understand (Supplementary Table S1). We evaluated the strengths and limitations of the responses provided by ChatGPT in the specific areas of pre-travel advice (Table 1) from the perspective of experienced and certified travel medicine specialists.

**Table 1:** Summary of potential strengths and weakness in the use of ChatGPT for pre-travel consultation and advice

**Questions**	**Strengths**	**Limitations**
*General travel advice* Should I seek a pre-travel clinic consultation prior to travelling? How can I avoid falling sick overseas? What precautions do I have to take for food and water safety overseas? Are there recommended additional precautions with regards to sexual health overseas? What can I take for traveller’s diarrhoea? How can I take precautions against altitude sickness?	Information provided is generally concise and accurate, given in point-form for ease of reading Appropriately and consistently recommends consultation with healthcare provider or travel clinic in addition to the specific advice given Gives accurate advice on food and water safety, safe sex, traveller’s diarrhoea and altitude sickness	Generic advice is not contextualized to the specific details of the travel itinerary Also does not take into account the specific details of the traveller’s background and medical comorbidities Gives both ciprofloxacin and azithromycin as options for traveller’s diarrhoea which may not be appropriate in all regions of travel
*Vaccinations* When should I get my pre-travel vaccines? Can I take my vaccinations if I am on high-dose steroids? Can I take the yellow fever vaccine if I am on steroids? What precautions do I need for travel to Mecca for the Haj? Is the meningococcal vaccine required for Haj pilgrimage? What vaccines are required for travel to Bogota, Columbia? What are some risks associated with vaccinations? What are some risks associated with Yellow Fever vaccination? Do I require COVID-19 vaccination for travel?	Appropriately and consistently advises further discussion with a healthcare provider or travel clinic Recommends the appropriate timing for vaccinations and also explains the potential utility in specific settings for last-minute vaccinations Appropriately identifies and explains the need for Yellow Fever vaccination in at-risk areas.	Does not mention meningococcal vaccine specifically for Haj, unless prompted Explanation for the vaccine-associated viscerotropic and neurotropic disease as part of the Yellow Fever vaccination is not clear as to the nature of the complication, and does not weigh it against the risk of acquiring Yellow Fever. Also does not mention COVID-19 precautions unless prompted, with information up-to-date only up to September 2021.
*Malaria prevention prophylaxis* How can I avoid mosquitoes? Do I need malaria prophylaxis for travel to Nairobi, Kenya? What prophylaxis for malaria is recommended for travel to Abidjan? What are the options I can use for malaria prophylaxis?	Gives appropriate advice for vector avoidance. Appropriately recognises the need for malaria prophylaxis in Kenya, if in line with the nature of the travel Gives all the appropriate options for malaria prophylaxis, as well as how they are to be taken, and common side effects	Does not take into account the individual’s potential co-morbidities in relation to options malaria chemoprophylaxis Did not consider geographic considerations and rates of resistance in the choice of malaria prophylaxis Also does not compare the cost of each option

In the first area of general travel queries, ChatGPT answered the questions accurately, although the advice provided was generic and not contextualized to the individual’s specific travel itinerary and medical co-morbidities. For example, a patient with a history of splenectomy would be at a significantly elevated risk of severe malaria, a vital consideration to highlight in the pre-travel consultation. ChatGPT also suggested ciprofloxacin or azithromycin for traveller’s diarrhoea. In most geographic areas, quinolone resistance is high and azithromycin may be the better option.^10^ The advice provided while logical, like choosing restaurants with ‘good hygiene practices’, may not necessarily be practical, as travellers cannot feasibly inspect a restaurant’s hygiene practices prior to making dining choices.

ChatGPT accurately gave advice for altitude sickness and the necessary precautions but similarly did not explain potential adverse effects of acetazolamide. Furthermore, while ChatGPT mentions the Andes and Himalayas, it fails to specifically mention Kilimanjaro as an important location for altitude-related fatality. In contrast, a human provider would have the ability to probe with follow-up questions and thus give more specific and detailed travel advice.

ChatGPT was able to provide an exhaustive list of important immunisations required for specific areas of travel, including the timing of these vaccines prior to travel (Supplementary Table S1 available as Supplementary data at JTM online, Table 1). For example, Bogota, Columbia was appropriately identified as an area not at high risk for yellow fever transmission, but that vaccination may still be recommended for travel to other parts of Columbia.^10^ Importantly, ChatGPT failed to emphasize that some vaccinations are required, such as the meningococcal vaccination for pilgrims going on Hajj. ChatGPT also does not readily take into account an individual’s prior immunization status and medical comorbidities in its recommendation. However, if prompted directly, it is able to provide appropriate advice. For example, when asked about yellow fever vaccination (which is a live vaccine) in an individual on steroids, ChatGPT appropriately raises concern, and directs the traveller to a healthcare provider for assessment.

Perhaps because recent outbreaks such as COVID-19 and human Mpox are not classically part of pre-travel advice, these vaccinations are also not specifically addressed, unless directly asked. ChatGPT also specifies in its reply that the information provided on COVID-19 is from September 2021, and does not include up-to-date recommendations.

Finally, with regards to malaria prevention, ChatGPT appropriately identifies geographical areas at risk for malaria transmission (we tested this on both Nairobi and Abidjan), provides comprehensive details on aspects of vector avoidance for travellers and lists options for malaria chemoprophylaxis (Table 1, Supplemental Table S1 available as Supplementary data at *JTM* online). It does not take into account an individual’s medical co-morbidities, or the geographic area of travel, which may influence choice of prophylaxis. Costs of various drugs are also often a very important consideration for travellers but are not addressed by ChatGPT. ChatGPT also fails to consider the specific nature of a patient’s travel. For example, a traveller who is largely staying indoors, and within the city centre of Nairobi may safely choose to decline malaria chemoprophylaxis.

The accessibility of ChatGPT at any time makes it easy for the layperson to use, and may improve the uptake of pre-travel advice and consultation. The advice provided may be more comprehensive and exhaustive than a human provider. For example, in preventing malaria, an option to reduce risk may be to choose to travel in the dry season instead of wet season. ChatGPT recognizes this and provides this advice, but a human provider may have neglected to consider that changing the date of travel may be an option.

There are important limitations to our findings and study design. It was designed as a conversation to explore commonly asked questions. Therefore, it was not an exhaustive evaluation of all potentially erroneous information that ChatGPT could provide. Furthermore, we only included a single AI chatbot, with no replication of experimental queries. ChatGPT also was not able to provide verifiable sources from which the information had been obtained, thus making it difficult for travellers to independently verify the accuracy of the responses.

Overall, the use of AI chatbots for pre-travel advice may be vital and complementary to the physical consultation. ChatGPT consistently highlights the importance of seeking advice from a healthcare provider prior to travel. Unlike a human provider, ChatGPT is accessible at any time, and provides comprehensive answers. It may at times lack specificity in contextualizing advice to the individual traveller and circumstance; however, a human healthcare provider could step in to fill that gap.

## Supplementary Material

TravelConsult_AI_SupplementalTable1_taad124

## Data Availability

Data may be made available on request from the corresponding author.

## Credit Author statement

Jinghao Nicholas Ngiam (Conceptualization-Equal, Data curation-Equal, Formal analysis-Equal, Writing—original draft [Equal]), Matthew Koh (Conceptualization-Equal, Data curation-Equal, Formal analysis-Equal, Writing—original draft [Equal]), Priscillia Lye (Conceptualization-Equal, Data curation-Equal, Formal analysis-Equal, Writing—original draft [Equal]), Tze Sian Liong (Conceptualization-Equal, Data curation-Equal, Formal analysis-Equal, Writing—original draft [Equal]), Brenda Salada (Conceptualization-Equal, Formal analysis-Equal, Writing—review & editing [Equal]), Paul Tambyah (Conceptualization-Equal, Formal analysis-Equal, Writing—review & editing [Equal]), and Jolene Oon (Conceptualization-Equal, Formal analysis-Equal, Writing—review & editing [Equal]).
