# Supplementary material for: Artificial intelligence models for pre-travel consultation and advice: yea or nay?
Source: J Travel Med. 2023 Oct 3;31(1):taad124. doi: 10.1093/jtm/taad124 (PMC10823483; doi:10.1093/jtm/taad124)
Supplement: TravelConsult_AI_SupplementalTable1_taad124 [file travelconsult_ai_supplementaltable1_taad124.docx]

**Supplementary Table 1: ChatGPT responses to the specific questions on pre-travel advice**

| **Questions and Responses** |
| --- |
| *General travel advice*  **Q: Should I seek a pre-travel clinic consultation prior to travelling?**  A: Consulting a pre-travel clinic can be beneficial, especially if you're traveling to regions with specific health risks. They can provide advice on vaccinations, preventive medications, and health precautions based on your destination and individual health profile. It's a good idea to consider a consultation, especially if you have any underlying health conditions or if your trip involves high-risk areas.  **Q: How can I avoid falling sick overseas?**  A: To reduce the risk of falling sick while traveling overseas, it's important to take a combination of preventive measures to protect your health. Here are some tips to help you stay healthy during your travels:  Vaccinations: Make sure you are up-to-date on routine vaccinations and any recommended travel vaccinations for your destination. Consult a travel clinic or healthcare provider for personalized advice.  Food and Water Safety: Drink bottled water or use water purification methods (boiling, filtering, or using water purification tablets).  Avoid consuming ice, tap water, or beverages made with tap water.  Eat cooked, hot, and freshly prepared foods. Avoid raw or undercooked foods.  Hand Hygiene: Wash your hands frequently with soap and clean water for at least 20 seconds, especially before eating or touching your face.  Carry hand sanitizer for times when soap and water are not available.  Safe Eating Practices: Choose restaurants and food vendors with good hygiene practices and clean facilities.  Peel fruits and vegetables or choose items that have been cooked.  Insect Protection: Use insect repellent on exposed skin and clothing to prevent insect-borne diseases like malaria, dengue, and Zika.  Wear long sleeves, pants, and socks in areas where insects are a concern.  Sun Protection: Wear sunscreen with a high SPF and reapply it regularly, especially if you're in sunny or tropical destinations.  Wear protective clothing and a hat to shield yourself from the sun.  Rest and Hydration: Get enough rest to maintain your immune system.  Stay hydrated by drinking plenty of fluids, especially in hot or humid climates.  Climate Adaptation:  Gradually acclimate to changes in climate and altitude, if applicable.  Travel Insurance: Purchase comprehensive travel insurance that includes medical coverage to ensure you're protected in case of illness or injury.  Personal Health: Carry any necessary medications and a small first aid kit.  Be mindful of your own health and any symptoms you may experience. Seek medical attention if needed.  Local Health Alerts: Stay informed about health advisories and disease outbreaks in your destination.  Cultural Considerations: Respect local customs and practices, especially related to food and hygiene.  Remember that while these precautions can help minimize your risk, it's not always possible to avoid all health risks while traveling. Being informed, practicing good hygiene, and taking care of your health can significantly increase your chances of having a safe and enjoyable trip.  **Q: What precautions do I have to take for food and water safety overseas?**  A: When it comes to food and water safety overseas, here are some precautions to consider:  Drink Safe Water: Stick to bottled water or water that has been properly treated and purified. Avoid tap water, ice cubes, and beverages made with tap water.  Eat Cooked Foods: Opt for hot, freshly cooked meals. Cooking at high temperatures can kill harmful bacteria. Avoid raw or undercooked seafood, meat, and eggs.  Peel It or Boil It: Eat fruits and vegetables that you can peel or have been cooked. Avoid salads or items that may have been washed with contaminated water.  Avoid Street Food: While street food can be tempting, it may not always meet the same hygiene standards as established eateries.  Check for Cleanliness: Choose restaurants and food vendors that appear clean and well-maintained. Good hygiene practices reduce the risk of foodborne illnesses.  Wash Hands: Always wash your hands with soap and clean water before eating. Carry hand sanitizer for times when clean water is not available.  Avoid Tap Water: Don't use tap water for brushing teeth. Use bottled or purified water for all oral hygiene practices.  Use Bottled Water for Drinks: When ordering beverages, ask for them without ice and ensure they are made with bottled or purified water.  Be Cautious with Dairy: Avoid unpasteurized dairy products, as they can carry harmful bacteria.  Stay Informed: Research local food and water safety recommendations for your specific destination before you travel.  Remember, each destination may have unique challenges, so tailor your precautions accordingly. It's always a good idea to consult with a travel clinic or medical professional for advice specific to your trip.  **Q: Are there recommended additional precautions with regards to sexual health overseas?**  A: Certainly, sexual health is an important aspect to consider while traveling overseas. Here are some additional precautions to keep in mind:  Safe Sex Practices: Just like at home, practicing safe sex is crucial. Use condoms consistently and correctly to reduce the risk of sexually transmitted infections (STIs) and unintended pregnancies.  Carry Your Own Supplies: Consider bringing your own condoms and other barrier methods from a trusted source to ensure their quality.  Avoid Risky Behaviors: Be cautious with casual sexual encounters. Engaging in high-risk sexual behaviors increases the chances of exposure to STIs.  Know Local Laws and Customs: Understand the local attitudes towards sex and sexual health. In some countries, certain sexual activities may be illegal or socially unacceptable.  Seek Medical Help: If you have concerns about sexual health or experience any symptoms of STIs, seek medical assistance promptly. Familiarize yourself with local healthcare resources.  Stay Informed: Research the prevalence of different STIs in your destination and any specific health advisories related to sexual health.  Consider Vaccinations: Some destinations may have higher rates of certain STIs. Check if there are recommended vaccines available for diseases like Hepatitis B.  Stay Hygienic: Practicing good personal hygiene can help reduce the risk of infections.  Travel Health Insurance: Ensure your travel insurance covers potential medical expenses related to sexual health issues.  Consult a Healthcare Professional: If you have specific concerns or questions about sexual health while traveling, consult a healthcare provider or a travel clinic.  Remember that your health and well-being are important. Taking precautions and being informed can help you have a safer and more enjoyable travel experience.  **Q: What can I take for traveller’s diarrhoea?**  A: Traveler's diarrhea is a common concern for those traveling to different countries, especially regions with different hygiene standards. To manage traveler's diarrhea, here are some steps and medications you can consider:  Hydration: The most important aspect of managing traveler's diarrhea is to stay hydrated. Drink plenty of clean, safe fluids, such as bottled water, oral rehydration solutions, clear broths, and diluted fruit juices.  Rest and Diet: Give your stomach some rest by avoiding solid foods initially. Gradually reintroduce bland, easy-to-digest foods like rice, bananas, plain yogurt, and toast.  Oral Rehydration Solutions (ORS): These solutions contain electrolytes and sugars that help replenish lost fluids and electrolytes due to diarrhea. They are available over-the-counter and can be mixed with safe drinking water.  Anti-Diarrheal Medications: Over-the-counter medications like loperamide (Imodium) can help reduce diarrhea symptoms and slow down bowel movements. However, these should be used cautiously and only as needed, as they can delay the elimination of infectious agents from your system.  Antibiotics: In some cases, if diarrhea is severe, persistent, or accompanied by fever, blood in stool, or other concerning symptoms, a healthcare provider may prescribe antibiotics. Common options include ciprofloxacin or azithromycin. However, antibiotics should only be used under medical supervision.  Probiotics: Some travelers find that taking probiotics before and during their trip can help maintain gut health and reduce the risk of traveler's diarrhea. Consult a healthcare provider before starting any supplements.  Remember, it's important to exercise caution when using medications and to consult a healthcare provider, especially if you have underlying health conditions, are taking other medications, or are traveling with children or elderly individuals. Additionally, it's crucial to practice good hygiene, such as washing your hands frequently, avoiding tap water and uncooked foods, and practicing safe food handling, to reduce the risk of traveler's diarrhea.  **Q: How can I take precautions against altitude sickness?**  A: Altitude sickness, also known as acute mountain sickness (AMS), can occur when you ascend to high altitudes too quickly, and your body doesn't have enough time to adjust to the lower oxygen levels. If you're traveling to a location with high altitudes, like areas in the Andes or the Himalayas, here are some precautions you can take to help prevent altitude sickness:  Gradual Ascent: Whenever possible, plan a gradual ascent to higher altitudes. Allow your body time to acclimate by spending a night or more at intermediate altitudes before reaching your final destination.  Stay Hydrated: Drink plenty of fluids, but avoid excessive alcohol and caffeine, which can contribute to dehydration. Proper hydration helps your body adjust to altitude changes.  Eat Light and Balanced: Opt for easily digestible and high-carbohydrate meals. Avoid heavy and fatty foods that can strain your digestive system.  Rest: Get plenty of rest during your first day or two at high altitudes. Avoid strenuous activities until your body has had a chance to adjust.  Avoid Overexertion: Engage in light activities initially and gradually increase your activity level as your body acclimates.  Stay Warm: Dress in layers to stay warm, as colder temperatures can exacerbate altitude-related discomfort.  Medications: Acetazolamide (Diamox) is a prescription medication that can help prevent and alleviate symptoms of altitude sickness. Consult a healthcare provider before using any medication and discuss the benefits and potential side effects.  Monitor Symptoms: Be aware of symptoms of altitude sickness, which can include headache, nausea, dizziness, fatigue, and shortness of breath. If symptoms worsen or become severe, descend to a lower altitude.  Oxygen: Some high-altitude locations provide supplemental oxygen, which can help alleviate symptoms. Portable oxygen canisters might also be useful, especially during the initial adjustment period.  Consult a Healthcare Provider: If you have any pre-existing medical conditions or concerns about altitude sickness, consult a healthcare provider before your trip. They can provide personalized advice and recommendations.  Remember that everyone's body reacts differently to altitude, and some individuals are more prone to altitude sickness than others. Taking precautions and allowing your body time to adjust is key to minimizing the risk of altitude sickness. If symptoms become severe or you're in doubt, it's always better to descend to a lower altitude and seek medical attention if necessary. |
| *Vaccinations*  **Q: When should I get my pre-travel vaccines?**  A: You should ideally start planning your pre-travel vaccinations well in advance, as some vaccines require multiple doses spaced over weeks or months to provide full protection. Here's a general timeline to consider:  4-6 Weeks Before Travel: Schedule a visit to a travel health clinic or healthcare provider at least 4-6 weeks before your departure. This allows time for any necessary vaccinations, as well as for the vaccines to take effect.  Review Your Immunization History: Before your appointment, gather information about your current immunization status. This will help the healthcare provider determine which vaccines you need.  Required and Recommended Vaccines: Discuss your travel plans with the healthcare provider. They will recommend vaccines based on your destination, activities, and individual health. Some vaccines may be required for entry into certain countries.  Multiple Doses: If you need vaccines that require multiple doses, such as Hepatitis A, Hepatitis B, or Typhoid, you may need to start the series well in advance to complete it before your trip.  Catch-Up Vaccinations: If you're not up-to-date on routine vaccinations (e.g., MMR, Tetanus), the healthcare provider may recommend catching up on those as well.  Yellow Fever Certificate: If you need a yellow fever vaccine, note that some countries require a yellow fever vaccination certificate for entry. You usually need to get the vaccine at an approved clinic and obtain an International Certificate of Vaccination or Prophylaxis (ICVP).  Health and Safety Precautions: In addition to vaccinations, discuss other health and safety precautions with your healthcare provider, such as insect repellents, antimalarial medications (if needed), and general travel health advice.  Last-Minute Travel: If you have limited time before your trip, still try to get any recommended vaccines, as some protection is better than none. However, some vaccines may not have enough time to take effect fully.  Remember that individual health factors and specific travel plans can affect the vaccine recommendations and timing. It's best to consult with a healthcare professional who specializes in travel medicine to create a personalized vaccination plan based on your needs and timeline.  **Q: Can I take my vaccinations if I am on high-dose steroids?**  A: If you are on high-dose steroids or other immunosuppressive medications, it's important to consult your healthcare provider or a specialist in travel medicine before getting vaccinations. The decision to receive vaccines while on immunosuppressive medications can be complex and requires careful consideration.  Here are a few points to keep in mind:  Risk and Benefit Assessment: Your healthcare provider will assess the potential risks and benefits of administering vaccines while you're on high-dose steroids. The decision will depend on factors such as the specific vaccine, your medical condition, the dosage of steroids, and your overall health.  Effectiveness of Vaccines: Immunosuppressive medications can sometimes weaken the immune response to vaccines, which might affect their effectiveness. Your healthcare provider will determine the appropriate timing and dosing of vaccines to maximize their benefit.  Vaccine Type: Some vaccines are considered safe for individuals on immunosuppressive medications, while others may pose a greater risk. Your healthcare provider will help you prioritize vaccines based on your travel plans and health status.  Weighing Risks: In some cases, the risk of vaccine-preventable diseases during travel might outweigh the potential risks associated with receiving vaccines while on immunosuppressive medications.  Alternative Precautions: If certain vaccines are not recommended while on immunosuppressive medications, your healthcare provider might recommend alternative precautions, such as avoiding high-risk areas or practicing enhanced hygiene measures.  Consultation: It's crucial to have a thorough discussion with your healthcare provider before making any decisions. They will consider your specific medical situation and tailor their recommendations accordingly.  Remember that this advice is general in nature, and individual circumstances can vary widely. Your healthcare provider will provide the most accurate and personalized guidance based on your medical history, medication regimen, and travel plans. It's essential to consult them before making any decisions about vaccinations while on high-dose steroids.  **Q: Can I take the yellow fever vaccine if I am on steroids?**  A: The decision to take the yellow fever vaccine while on steroids should be made in consultation with a healthcare professional. Certain medical conditions and medictions, including steroids, can affect your ability to receive vaccines. Steroids can weaken the immune system, potentially reducing the vaccine’s effectiveness. However, your healthcare provider can assess your individual health situation and advise you on the best course of action, including the timing of vaccinations and any precautions that may be necessary. Always seek personalized medical advice for such situations.  **Q: What precautions do I need for travel to Mecca for the Hajj?**  A: Traveling to Mecca for Hajj is a significant and religiously important journey. It's important to take various precautions to ensure a safe and spiritually fulfilling experience. Here are some important considerations:  Health Precautions: Consult a healthcare provider before traveling to ensure you're in good health and receive any necessary vaccinations.  Carry essential medications, first aid supplies, and personal hygiene items.  Stay hydrated and follow recommended food safety practices to prevent foodborne illnesses.  Pilgrimage Preparations: Familiarize yourself with the Hajj rituals and procedures in advance.  Carry necessary documentation, including your Hajj permit, passport, and identification.  Dress modestly and comfortably in accordance with Islamic traditions.  Safety and Security: Be aware of your surroundings and follow local laws and customs.  Keep your belongings secure and avoid displaying valuable items.  Stay informed about any security alerts or updates from local authorities.  Accommodations: Choose accommodations that are reputable, clean, and well-maintained.  Respect the rules and guidelines of your lodging and follow safety protocols.  Hygiene and Sanitation: Practice good hygiene, especially during crowded gatherings. Carry hand sanitizers and wash your hands frequently.  Be aware of sanitation facilities and use them appropriately.  Heat and Sun Protection: Mecca can have high temperatures, so wear appropriate clothing to protect yourself from the sun and stay hydrated.  Spiritual Preparedness: Engage in spiritual and mental preparation for the pilgrimage. Reflect on the significance of the journey and its religious importance.  Cultural Sensitivity: Respect local customs and etiquette. Be mindful of cultural norms and religious practices.  Communication: Keep in touch with your travel companions and loved ones. Share your travel itinerary and contact information.  Emergency Contacts:  Carry emergency contact information for local authorities, your embassy, and your Hajj tour group (if applicable).  Remember that Hajj is a unique and sacred experience, and the pilgrimage involves a significant amount of physical and emotional effort. Taking the necessary precautions will help you have a safe, meaningful, and spiritually enriching journey. It's also advisable to stay updated on any Hajj-related guidelines or advisories issued by the Saudi Arabian authorities.  **Q: Is the meningococcal vaccine required for Hajj pilgrimage?**  A: Yes, the meningococcal vaccine (specifically the quadrivalent meningococcal vaccine, which protects against four types of the bacteria) is a requirement for obtaining a Hajj visa to Saudi Arabia. This vaccination is mandated by the Saudi Arabian government as a preventive measure to reduce the risk of meningococcal disease transmission during the annual Hajj pilgrimage.  The Saudi authorities have specific vaccination requirements for Hajj, and proof of vaccination is usually checked upon entry into the country. Meningococcal disease can spread easily in crowded settings, making it a concern during large gatherings like the Hajj.  It's important to check with your healthcare provider or travel clinic to ensure that you have the appropriate vaccinations and documentation before embarking on your Hajj journey. They will provide you with the necessary information and ensure that you meet all the health and vaccination requirements set by Saudi Arabia.  **Q: What vaccines are required for travel to Bogota, Columbia?**  A: For travel to Bogotá, Colombia, there are several recommended and sometimes required vaccinations. These recommendations may vary based on your individual health status, the duration of your stay, and the specific activities you plan to engage in. It's important to consult a travel health clinic or healthcare provider for personalized advice. However, here are some vaccines that are commonly recommended for travelers to Colombia:  Routine Vaccinations: Make sure your routine vaccinations are up-to-date. These may include vaccines like measles-mumps-rubella (MMR), diphtheria-tetanus-pertussis (DTaP), polio, and influenza.  Hepatitis A: This vaccine is recommended for most travelers to Colombia, as Hepatitis A can be transmitted through contaminated food and water.  Typhoid: Typhoid fever can also be contracted through contaminated food and water. The vaccine is recommended for travelers to areas with poor sanitation.  Yellow Fever: While Bogotá itself is not a high-risk area for yellow fever, the vaccine might be required if you plan to visit other parts of Colombia. Check if you need a yellow fever vaccination based on your itinerary.  Hepatitis B: Depending on your activities and potential exposure (such as medical procedures or intimate contact), your healthcare provider might recommend the Hepatitis B vaccine.  Rabies: If you plan to engage in outdoor activities or have a higher risk of animal exposure, consider discussing the rabies vaccine with a healthcare professional.  Malaria: While Bogotá is generally considered a low-risk area for malaria, other parts of Colombia might have a higher risk. Consult a healthcare provider about malaria prevention measures, including medication, if you plan to travel to higher-risk areas.  Other Vaccines: Depending on your travel plans, your healthcare provider might recommend additional vaccines such as meningococcal, varicella (chickenpox), or others.  Keep in mind that vaccination requirements and recommendations can change, so it's best to consult with a travel health clinic or your healthcare provider closer to your travel date. They can provide you with the most up-to-date and personalized information based on your specific circumstances.  **Q: What are some risks associated with vaccinations?**  A: Vaccinations are generally safe and effective, but like any medical intervention, they can carry some risks. It's important to note that the risks of not getting vaccinated often far outweigh the potential risks associated with vaccination. Here are some potential risks associated with vaccinations:  Mild Side Effects: Common side effects of vaccines can include pain or swelling at the injection site, low-grade fever, fatigue, and mild discomfort. These side effects are usually short-lived and resolve on their own.  Allergic Reactions: Serious allergic reactions to vaccines are extremely rare. However, some individuals may have allergies to certain vaccine components. Vaccine providers are trained to manage allergic reactions if they occur.  Adverse Events: In very rare cases, vaccines can cause more serious adverse events, such as severe allergic reactions (anaphylaxis) or other rare side effects. These events are exceedingly uncommon and are closely monitored by healthcare professionals.  Guillain-Barré Syndrome (GBS): In rare instances, certain vaccines have been associated with an increased risk of Guillain-Barré Syndrome, a neurological disorder. The risk of GBS from vaccines is very low compared to the risk of developing GBS from a natural infection.  Febrile Seizures: Some vaccines, particularly in young children, can lead to a temporary high fever that might trigger a febrile seizure. These seizures are usually brief and do not typically have lasting effects.  Syncope (Fainting): Fainting can occur after any medical procedure, including vaccinations. Vaccine providers typically recommend sitting or lying down for a short period after receiving a vaccine to reduce the risk of fainting.  It's important to emphasize that the vast majority of people who receive vaccines experience no serious side effects. The benefits of vaccination, including the prevention of serious and potentially life-threatening diseases, far outweigh the risks. Before receiving any vaccine, it's a good practice to discuss any concerns or questions you have with your healthcare provider. They can provide you with accurate information about the specific vaccine, its benefits, and potential risks based on your individual health status.  **Q: What are some risks associated with Yellow Fever vaccination?**  A: The yellow fever vaccine is generally safe and effective, but like any vaccine, it can carry some risks. The risks associated with the yellow fever vaccine are relatively rare, and the vast majority of people who receive the vaccine experience no serious side effects. Here are some potential risks associated with the yellow fever vaccine:  Local Reactions: Mild side effects at the injection site are common and may include pain, redness, or swelling. These symptoms are usually mild and temporary.  Fever: Some people may experience a low-grade fever within a few days of receiving the vaccine. Fever is usually mild and short-lived.  Allergic Reactions: Serious allergic reactions to the yellow fever vaccine are extremely rare. However, as with any vaccine or medication, there is a very small risk of severe allergic reactions (anaphylaxis).  YEL-AVD: Yellow Fever Vaccine-Associated Viscerotropic Disease (YEL-AVD) is an extremely rare and serious side effect. It involves an immune response that affects various organs. YEL-AVD occurs at an estimated rate of about 1 in 1 million doses administered.  YEL-AND: Yellow Fever Vaccine-Associated Neurologic Disease (YEL-AND) is another rare adverse event that affects the nervous system. The risk is estimated to be around 1 in 2.4 million doses.  Risk Groups: Some individuals, such as those with weakened immune systems or severe allergies to vaccine components, may be at a higher risk of experiencing adverse events.  Before receiving the yellow fever vaccine, it's important to discuss any concerns or questions you have with your healthcare provider. They can provide you with accurate information about the vaccine, its benefits, and potential risks based on your individual health status. If you're planning to travel to an area where yellow fever is endemic or required for entry, the benefits of receiving the vaccine generally outweigh the risks, especially in areas with high risk of yellow fever transmission.  **Q: Do I require COVID-19 vaccination for travel?**  A: As of my knowledge cutoff date in September 2021, requirements for COVID-19 vaccination for travel vary widely depending on the destination, the airline, and the specific travel regulations in place. Some countries may require proof of COVID-19 vaccination as a condition of entry, while others may have different entry requirements such as negative COVID-19 tests, quarantine measures, or a combination of these.  It's important to keep in mind that COVID-19 travel requirements are subject to change, and new regulations may be implemented as the situation evolves. Before you travel, I recommend checking with the official government websites of your destination country and the airline you're flying with for the most up-to-date information on entry requirements, including vaccination and testing.  Additionally, your own vaccination status and any travel advisories from your home country's health authorities should be taken into consideration. If you have specific travel plans, it's advisable to consult with your healthcare provider or a travel medicine specialist to get personalized advice based on your individual circumstances.  Please note that the COVID-19 situation and travel requirements may have changed since my last knowledge update in September 2021. It's important to stay informed about the latest developments and guidelines related to COVID-19 and travel from reliable and official sources. |
| *Malaria prophylaxis*  **Q: How can I avoid mosquitoes?**  A: Avoiding mosquitoes is important to reduce the risk of mosquito-borne diseases such as malaria, dengue, Zika virus, and more. Here are some tips to help you avoid mosquitoes while traveling:  Use Insect Repellent: Apply an effective insect repellent to exposed skin and clothing. Look for repellents containing DEET, picaridin, or oil of lemon eucalyptus. Follow the product instructions for proper application.  Wear Protective Clothing: Wear long-sleeved shirts, long pants, socks, and closed-toe shoes to minimize exposed skin.  Choose light-colored clothing, as mosquitoes are often attracted to dark colors.  Stay Indoors: During peak mosquito activity times (dawn and dusk), try to stay indoors or in well-screened areas.  Sleep Under Mosquito Nets: If you're in an area with a high risk of mosquito-borne diseases, sleep under a bed net treated with insecticide. Make sure the net is properly tucked in and has no holes.  Use Air Conditioning: Mosquitoes are less likely to enter air-conditioned rooms. If available, use air conditioning or fans to keep your accommodation cool.  Eliminate Breeding Sites: Mosquitoes breed in standing water. Empty or cover containers that can collect water, such as flower pots, buckets, and discarded tires.  Keep gutters and drains clear to prevent water accumulation.  Avoid Fragrances: Avoid using strongly scented lotions, soaps, and perfumes, as they can attract mosquitoes.  Stay Away from Marshy Areas: If possible, avoid spending time near marshes, swamps, and areas with stagnant water, which are breeding grounds for mosquitoes.  Use Bed Netting: If you're camping or sleeping outdoors, use a mosquito-proof tent or mosquito netting.  Keep Doors and Windows Closed: Make sure doors and windows have screens to keep mosquitoes out.  Treat Clothing: Consider treating your clothing with permethrin, an insect repellent that can be sprayed on clothing or purchased pre-treated.  Travel During Dry Season: If feasible, plan your travel during the dry season when mosquito populations are generally lower.  Remember that while these measures can help reduce your risk of mosquito bites, it's not always possible to avoid them entirely. Being diligent in practicing these precautions will significantly reduce your exposure to mosquitoes and the potential for mosquito-borne illnesses.  **Q: Do I need malaria prophylaxis for travel to Nairobi, Kenya?**  A: Nairobi, the capital city of Kenya, is situated at a higher elevation and is considered a low-risk area for malaria. The risk of contracting malaria in Nairobi is generally lower compared to other parts of Kenya that are at lower altitudes and have higher mosquito activity.  However, while the risk is lower, it's not completely absent. It's recommended to take precautions to prevent mosquito bites, such as using insect repellents, wearing long sleeves and pants, and staying in air-conditioned or well-screened accommodations. If you plan to explore other parts of Kenya at lower altitudes where malaria risk is higher, you should consider malaria prophylaxis.  For specific recommendations about malaria prophylaxis and other travel health advice, I strongly recommend consulting with a travel medicine specialist or healthcare provider. They can provide personalized guidance based on your travel plans, medical history, and individual risk factors. It's important to make an informed decision about malaria prevention measures before your trip.  **Q: What prophylaxis for malaria is recommended for travel to Abidjan?**  A: I recommend checking the latest travel health recommendations from a reliable source like the CDC or a travel clinic, as they can provide up-to-date information on malaria prophylaxis for specific regions, including Abidjan. Malaria prevention measures often include anti-malarial medications, insect repellent, and bed nets. The choice of medication and specific recommendations may depend on factors like your health, the time of year you’re traveling, and the duration of your stay. Consulting a healthcare professional for personalized advice is essential.  **Q: What are the options I can use for malaria prophylaxis?**  A: There are several options for malaria prophylaxis (preventive medications) that you can discuss with a healthcare provider or travel medicine specialist. The choice of prophylaxis depends on factors such as the specific destination, your health status, potential side effects, and any contraindications. Here are some common options:  Atovaquone-Proguanil (Malarone): This is a combination medication that is taken daily, starting a day before entering a malaria-risk area and continuing for a week after leaving. It's generally well-tolerated but can be more expensive.  Doxycycline: This antibiotic is taken daily, starting a day before entering a malaria-risk area and continuing for four weeks after leaving. It's an economical option but can cause sun sensitivity and gastrointestinal side effects in some people.  Mefloquine (Lariam): This medication is taken once a week, starting at least two to three weeks before entering a malaria-risk area and continuing for four weeks after leaving. It's effective but can have potential neuropsychiatric side effects.  Chloroquine or Hydroxychloroquine: These medications were commonly used for malaria prevention, but their effectiveness is limited in many parts of the world due to increasing drug resistance.  Primaquine: This medication is used to prevent relapse of malaria caused by the Plasmodium vivax parasite. It's typically used in addition to other prophylactic medications for specific areas where this parasite is prevalent.  Tafenoquine: Similar to primaquine, tafenoquine is used for preventing relapse of Plasmodium vivax malaria and may be taken weekly.  It's important to note that each medication has specific dosing instructions, potential side effects, and contraindications. Additionally, malaria prophylaxis is just one aspect of preventing malaria. You should also take measures to avoid mosquito bites, such as using insect repellents, wearing protective clothing, and sleeping under mosquito nets.  Consult with a healthcare provider or travel medicine specialist to determine the most suitable malaria prophylaxis option for your specific travel plans and medical history. They can provide personalized recommendations and help you make an informed decision. |
